# Performance and Microstructure of Cold Recycled Mixes Using Asphalt Emulsion with Different Contents of Cement

**DOI:** 10.3390/ma12162548

**Published:** 2019-08-10

**Authors:** Yanhai Yang, Ye Yang, Baitong Qian

**Affiliations:** School of Transportation Engineering, Shenyang Jianzhu University, Shenyang 110168, China

**Keywords:** reclaimed asphalt pavement, cold recycled mixes, asphalt emulsion, performance, microstructure

## Abstract

Cold recycled mixes using asphalt emulsion (CRME) is an economical and environmentally-friendly technology for asphalt pavement maintenance and rehabilitation. In order to determine the optimum range of cement contents, the complex interaction between cement and asphalt emulsion and the effects of cement on performance of CRME were investigated with different contents of cement. The microstructure and chemical composition of the fracture surface of CRME with different contents of cement were analyzed in this paper as well. Results show that the high-temperature stability and moisture susceptibility of CRME increased with the contents of cement increasing. The low-temperature crack resistance ability gradually increased when the content of cement is increased from 0% to 1.5%. However, it gradually decreased when the content of cement is increased from 1.5% to 4%. Cold recycled mixes had better low-temperature cracking resistance when the contents of cement were in the range from 1% to 2%. The results of microstructure and energy spectrum analysis show that the composite structure is formed by hydration products and asphalt emulsion. The study will be significant to better know the effects of cement and promote the development of CRME.

## 1. Introduction

According to recent data, about 60 million tons of old asphalt materials are produced in China every year. These materials occupy a mass of land and pollute the environment. In addition, the shortage of suitable property aggregates severely limits the construction and maintenance in the pavement industry, which causes resource and environmental problems [1]. Highway agencies have attempted to reuse old asphalt materials to solve these critical issues [2]. One of the promising ways to recycle old asphalt materials is cold recycling technology [3]. The cold recycling technology has been largely used as surface and base course in a pavement system [2,4], which can make full use of plenty of old asphalt materials and reduce environmental pollution from old asphalt materials and greenhouse gases [5]. Since the 1970s, asphalt emulsion has been used as the most common recycling agent in cold recycled mixes because it is liquid at room temperature and helps the dispersion of asphalt emulsion in the mix.

However, with the earlier studies and experiences of cold recycled mixes using asphalt emulsion (CRME), some problems appeared, such as excessive deformation, low initial strength, and weak adhesiveness [3]. Therefore, many additives, such as fly ash, cement, and limestone powder, were used to enhance the performance of CRME [6,7]. However, additives are not the only way to improve the performance of CRME. The modified asphalt emulsion could also be considered as another means [8]. Compared to other additives the cement has better effects [9,10]. Generally, the content of cement is from 1 wt.% to 3 wt.% in CRME [11]. CRME might be classified as asphalt-stabilized material when the content of cement is lower. In contrast, CRME might be classified as cement-treated material when the content of cement is greater [12]. The main effect of cement on the performance is controlled by the reactions between cement and asphalt emulsion. The cement not only could accelerate the emulsion breaking, but can also increase the stiffness of CRME [13]. Moreover, hydration products could enhance the adhesion between the mortar and aggregates with an increase in the amount of cement [14]. Meanwhile, the strength development mechanism of CRME containing cement has been explained based on image analysis. The adhesive failure in the interface between emulsion-cement mortar and the aggregate could be considered as the dominant type of fracture at the early stage of curing. The main type of failure was a cohesive fracture within asphalt emulsion mortar after curing [15].

Cement plays a positive role on the physical properties of CRME [16]. Cement (especially in high dosages) could reduce the density of CRME [17]. The stiffness of CRME increases by increasing the cement dosage [18]. Meanwhile, cement could improve the moisture susceptibility [19,20] since hydration products increase the cohesiveness of CRME [21]. In addition, cement could enhance the mechanical properties of CRME [22,23]. Cement also could improve the resistance to traffic abrasive effect [24]. Normally, mechanical properties of CRME increase with the increase of curing time and cement content [25]. However, CRME could have a more brittle behavior under low temperature conditions when the content of cement surpasses a specific limit [26]. The fatigue behavior of CRME with cement has been evaluated based on an indirect tensile fatigue test. The cement reduces the fatigue life at strain levels of 300 microstrain or higher. In contrast, at strain levels less than 300 microstrain using cement the fatigue life increased [27].

The effects of cement on the performance of CRME have been reported in many studies. However, few studies have been conducted on performance and microstructure analysis of CRME whether cement is added or not. Therefore, this paper aims to look into the performance and microstructure of CRME at different contents of cement. The high-temperature deformation of CRME will decrease the road roughness. The low-temperature shrinkage cracking is the beginning of various pavement distresses. Because of the high air void (9–12%) of CRME, water easily could enter the internal mixture. Due to the evaporation of water in asphalt emulsion and the effect of cement in the mixing and compacting process of CRME, the cement emulsified asphalt mortar appears air void when the mortar is solidified completely [3,28]. Therefore, it is less cohesive and has difficulty repeatedly resisting the erosion of high-pressure water. So, the high-temperature stability, low-temperature shrinkage cracking resistance and moisture susceptibility of CRME should be evaluated. Then, the microstructure of CRME was examined by the use of scanning electron microscopy (SEM), which could observe the microstructure of fracture surfaces and analyze the chemical composition of mortar on CRME. The action mechanism of cement and asphalt emulsion was studied at the same time. Finally, the optimum cement content of CRME was learned through the analysis of performance and microstructure. This study will be significant to better know the effects of cement on and promote the development of CRME.

## 2. Materials and Methods

### 2.1. Materials

The CRME was made up of asphalt emulsion, reclaimed asphalt pavement (RAP), new aggregate, ordinary Portland cement, and water. Cationic slow-cracking asphalt emulsion was used in the research. The basic properties of asphalt emulsion are given in Table 1 based on JTG F41-2008 [29]. The RAP was collected from the surface of a first-class highway in Liaoning Province of China. The asphalt content of RAP is 4.5 wt.% The water content of RAP is 0.85 wt.% The gradation of RAP (in Table 2) could not meet gradation composition of cold recycled mixes in China. The new aggregates consisting of 16–19 mm, 13.2–16 mm, 9.5–13.2 mm and 2.36–4.75 mm were joined to meet the gradation, according to JTG F41-2008 [29]. The gradation of new aggregates is given in Table 3. Besides, the 32.5 of ordinary Portland cement was added. The initial setting time of the cement was 3.5 h. The final setting time of the cement was 6.7 h. The pre-mix water used in this experiment was from drinking water without any impurities.

### 2.2. Mix Design and Specimen Preparation

#### 2.2.1. Mix Design

**Figure 1:**
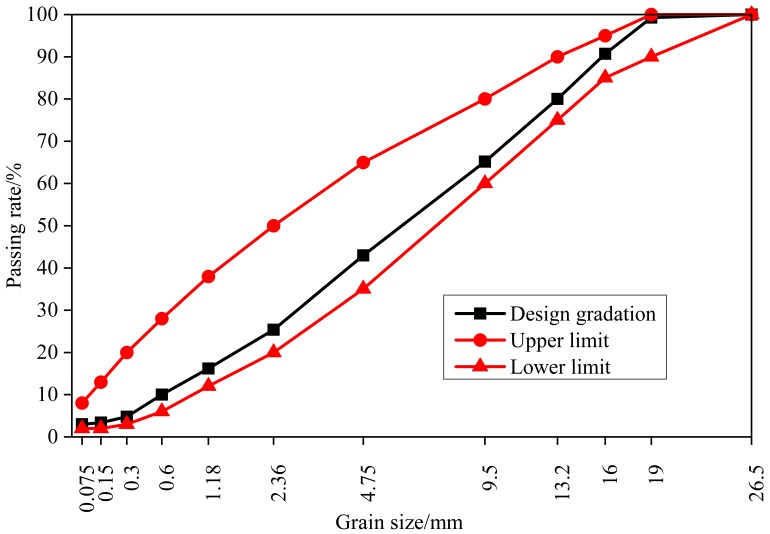
Grading curve of CRME.

The CRME was made up of RAP (71.5 wt.%) and new aggregate (28.5 wt.%). The design gradation consisted of RAP (71.5 wt.%), 16–19 mm (9 wt.%), 13.2–16 mm (10 wt.%), 9.5–13.2 mm (7 wt.%) and 2.36–4.75 mm (2.5 wt.%) in accordance with JTG F41-2008 [29]. The graduation curve was shown in Figure 1. The CRME was designed by the modified Marshall method based on JTG F41-2008 [29]. At first, the optimum water content of CRME was determined by maximum dry density in accordance with JTG E40-2007 [30]. Then, the optimum asphalt emulsion content of CRME was determined by air voids, Marshall stability and Marshall residual stability of Marshall specimens under the optimum water content in accordance with JTG F41-2008 [29]. The optimum content of asphalt emulsion and water of CRME were 3.5 wt.% and 4.3 wt.%.

#### 2.2.2. Specimen Preparation

The CRME specimens with different contents of cement (0%, 1%, 2%, 3%, 4%, 5%) were prepared under the optimum content of water and asphalt emulsion.

Marshall specimen preparation: Firstly, the CRME was put in the mold and compacted 50 blows per side with a Marshall hammer. Secondly, the whole compacted specimens were left in the mold and cured at 60 °C for at least 40 h in a draft oven. Thirdly, the whole CRME specimens were compacted 25 blows per side with a Marshall hammer immediately while they were taken out of the draft oven. Finally, the cured specimens were left to cool at room temperature for at least 12 h [29].

Slab specimen preparation: Firstly, cold recycled mixes were compacted into slab specimen by a steel roller. Then, the compacted slab specimens were cured at 60 °C for at least 40 h in a draft oven. Finally, the cured specimens were left to cool at room temperature for at least 12 h. The air void of slab specimens was equal to the air void of mix design after curing. The size of slab specimen was 300 mm × 300 mm × 50 mm [29,31].

Beam specimen preparation: The beam specimens were prepared by the slab specimens (300 mm × 300 mm × 50 mm) cutting after curing. The size of the beam specimens was 250 mm × 30 mm × 35 mm [31].

### 2.3. Experimental Methods

#### 2.3.1. High-Temperature Stability Test

The rutting test was carried out to evaluate the high-temperature stability of CRME. The rutting test was conducted at 60 ± 1 °C. The 0.7 MPa loading was performed on the specimen surface by a moving solid rubber tire with the speed of 42 ± 1 cycles/min in accordance with JTG E20-2011 [31]. Two indices of rut depth (RD) and dynamic stability (DS) were collected. The amount of rutting deformation was recorded at 45 min and 60 min. DS is calculated by loading times to rutting depth ratio.

#### 2.3.2. Low-Temperature Cracking Resistance Test

Three-point bending test was used to evaluate the low-temperature cracking resistance of CRME, which was conducted at −10 °C with a loading rate of 50 mm/min in accordance with JTG E20-2011 [31]. Asphalt mixture test system (LMT-2) (Changsha Yaxing Numerical Control Technology Co., Ltd., Changsha, China) was applied to carry out the low-temperature cracking resistance test. The failure strain was calculated by Equation (1). The failure strain was applied to assess the low-temperature performance of CRME.

*ε*_B_ = 6*hd*/*L*^2^ (1)

where, *ε*_B_, *h*, *d* and *L*, represent failure strain (με), specimen height (cm), failure deformation (cm), and specimen length (cm), respectively.

#### 2.3.3. Moisture Susceptibility Test

In this study, the freeze-thaw test was carried out to evaluate moisture damage of CRME at different contents of cement in accordance with JTG E20-2011 [31]. Twelve Marshall specimens were prepared and divided into two groups for the freeze-thaw test. The first group of six specimens stayed in 25 °C water for over 2 h before testing. The second group of six specimens need the following process before testing: (1) specimens were vacuumed for 15 min under 0.09 MPa pressure water and then stayed in water at ordinary pressure for 0.5 h; (2) specimens were placed in plastic bags with 10 mL water at −18 °C for 16 h; (3) specimens were put in 60 °C water bath for 24 h without the plastic bags; (4) specimens were kept in a 25 °C water bath for over 2 h. The percentage of the Indirect Tensile (IDT) strength ratio, Tensile Strength Ratio (TSR) (%), was obtained by Equation (2).

TSR = *R_T_*_1_/*R_T_*_2_ × 100 (2)

where, *R_T_*_1_ and *R_T_*_2_ represent the IDT strength of the second group (MPa), and the IDT strength of the first group (MPa), respectively.

#### 2.3.4. SEM Test and Energy Spectrum Analysis

Breaking the specimen after cooling, the 10 slices with the same cement content of specimen fracture surface mortar were taken into clean containers for observation to avoid the surface pollution of mortar. The phase composition and chemical composition of CRME fracture surface were observed through SEM (Hitachi Limited, Tokyo, Japan) and energy dispersive system by PHILIPS-FEI Quanta 200. The chemical elements above boron (B) could be analyzed by the energy dispersive system of SEM PHILIPS-FEI Quanta 200.

## 3. Results and Discussion

### 3.1. High-Temperature Stability

**Figure 2:**
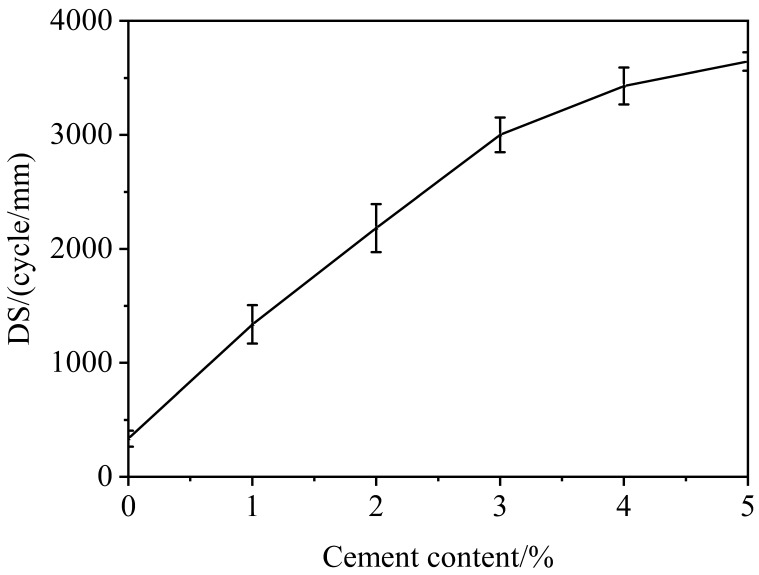
Results of rutting test.

As shown in Figure 2, the DS of CRME increases as cement content increases. On the one hand, hydration products and emulsion breaking form composite mortar which improves adhesive property between aggregate and mortar. On the other hand, hydration products are rigid material. Therefore, hydration products have good temperature stability and stiffness compared with asphalt mortar. Hydration products could improve temperature stability and deformation resistance ability of composite mortar. So, the high-temperature performance of CRME is enhanced. The DS of CRME increases significantly initially and then increases slowly when the content of cement is from 1% to 5%. The composite structure is formed rapidly by hydration products and asphalt emulsion when the content of cement is less than 3%, which fills the voids of the mixture. Consequently, the DS of CRME increases rapidly. Because the cement could not hydrate because of insufficient water when the content of cement is over 3%, the unhydrated cement only acts as filler. So, the DS increases slowly.

### 3.2. Low-Temperature Cracking Resistance

**Figure 3:**
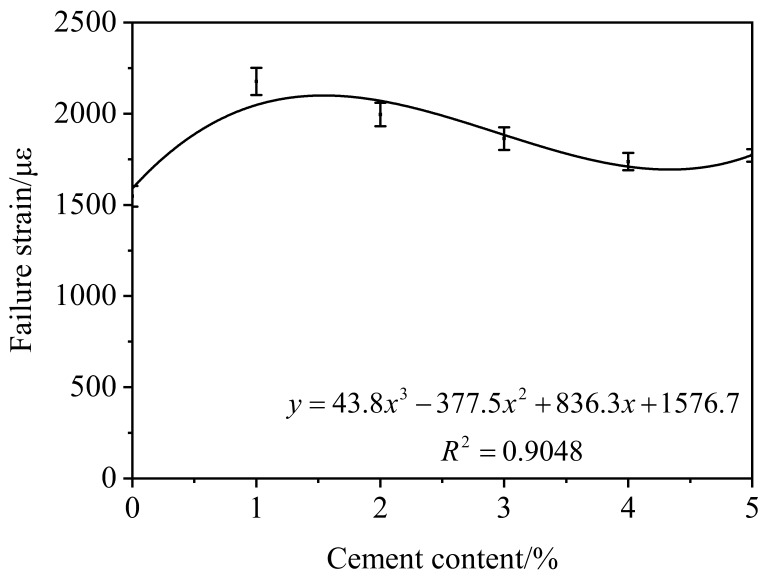
Results of three-point bending test.

As shown in Figure 3, the law of failure strain obeys cubic polynomial. The failure strain gradually increased when the content of cement is from 0% to 1.5%. However, it gradually decreased when the content of cement is from 1.5% to 4%. The failure strain reaches the maximum when the content of cement is about 1.5%. The results indicate the optimum content of cement may exist. Asphalt is viscous-elastic material and hydration products have high strength. Hydration products are coated by the asphalt emulsion. The composite structure is formed, which could enhance the low-temperature cracking resistance. Hydration products increase with cement content increasing. So the low-temperature cracking resistance is decreased. Cement could not hydrate because of insufficient water as the content of cement increases. The unhydrated cement only acts as filler. So the failure strain of CRME decreases slowly with the content of cement changing from 4% to 5%.

### 3.3. Moisture Susceptibility

**Figure 4:**
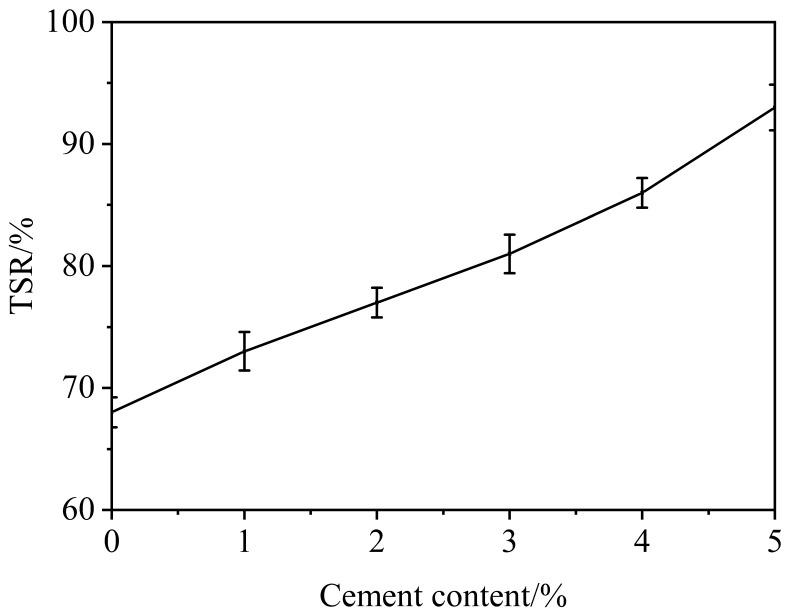
Results of moisture susceptibility test.

As shown in Figure 4, the TSR of CRME with different contents of cement is improved obviously. The composite structure is formed by hydration products and asphalt emulsion, which brings loose RAP together tightly. At the same time, the unhydrated cement of CRME would continue to hydrate when the freeze-thaw test of CRME is carried out. So the strength continues to increase at high cement content.

### 3.4. Microstructure Analysis

**Figure 5:**
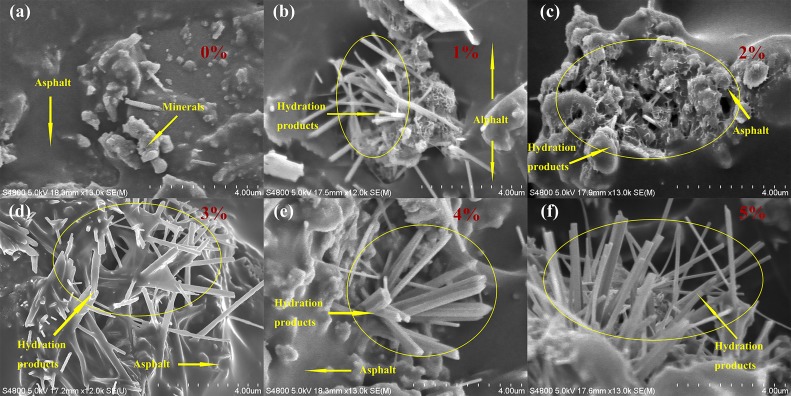
Microstructure of mortar with different contents of cement. (**a**) Without cement. (**b**) With 1% cement. (**c**) With 2% cement. (**d**) With 3% cement. (**e**) With 4% cement. (**f**) With 5% cement.

**Figure 6:**
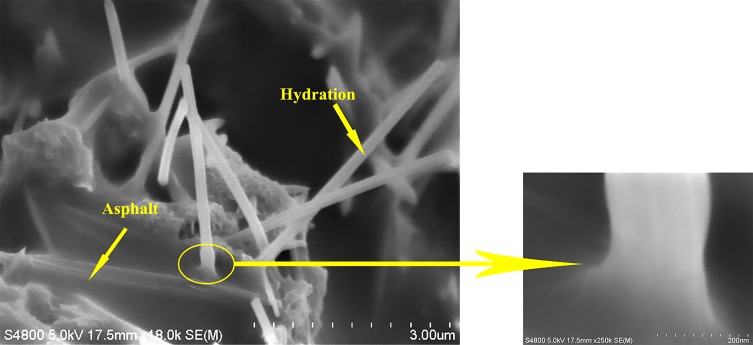
The adhesive state between asphalt membrane and hydration products.

The microstructure of mortar is shown in Figure 5 and Figure 6. As shown in Figure 5a, the surface of mortar was not smooth. However, hydration products were not observed in the surface of mortar. So the mortar was made up of asphalt and minerals. From Figure 5b–f, hydration products were observed in the surface of mortar when the content of cement was increasing from 1% to 5%. Nonetheless, as shown in Figure 5b,c, the proportion of hydration products was less when the content of cement was 1% and 2%. Moreover, it was a semi-wrapped state of asphalt and hydration products. From Figure 5d–f, the proportion of hydration products increased when the content of cement was increasing from 3–5%. Composite mortar was mainly composed by hydration products. The proportion of hydration products was dominant when the content of cement was 3–5%. The asphalt membrane was penetrated by hydration products. Meanwhile, Figure 6 illustrates that the connection between asphalt membrane and hydration products was continuous and smooth without cracks. It indicates that there was good adhesion between asphalt membrane and hydration products.

The main effect of cement on the performance of CRME is controlled by the proportion of hydration products and entrapment state between asphalt and hydration products. As shown in Figure 5b–d, by increasing the cement dosage from 1–5%, products of hydration process increase gradually. Hydration products are rigid material which can improve stiffness and temperature stability of CRME. Meanwhile, hydration products distributed in asphalt mortar could enhance adhesion between the mortar and aggregates (new aggregate and reclaimed asphalt pavement). Hence, the high-temperature stability and moisture susceptibility of CRME is enhanced with the cement contents from 1–5%. On the other hand, from Figure 5b,c, when composite mortar contains 1–2% cement dosage, the asphalt is dominant and hydration product is enwrapped by asphalt. CRME manufactured with composite mortar containing 1–2% cement is bitumen-stabilized materials, and its behavior is similar to asphalt mixes under low-temperature condition. However, composite mortar was mainly constituted by hydration products when the content of cement was 3–5%, as shown in Figure 5d–f. CRME manufactured with composited mortar containing 3–5% cement is cement-treated materials. At this time, CRME could have a more brittle behaviour under low-temperature condition. Generally speaking, CRME has good low-temperature cracking resistance ability when cement dosage is 1–2%.

### 3.5. Energy Spectrum Analysis

Ordinary Portland cement is mainly made up of 3Cao·SiO_2_ (C_3_S), 2CaO·SiO_2_ (C_2_S), 3CaO·Al_2_O_3_ (C_3_A), 4CaO·Al_2_O_3_·Fe_2_O_3_ (C_4_AF), CaSO_4_ and so on. Hydration occurs when the cement meets water.

**Figure 7:**
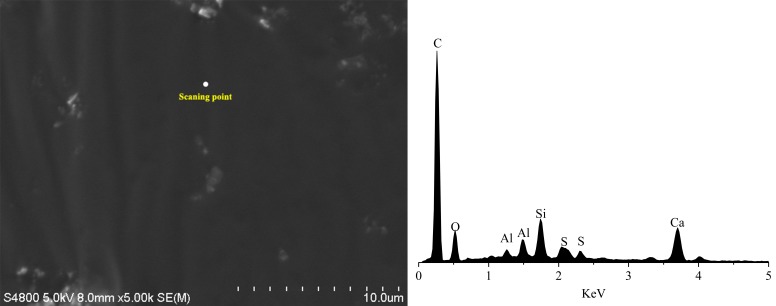
Chemical composition of CRME without cement.

**Figure 8:**
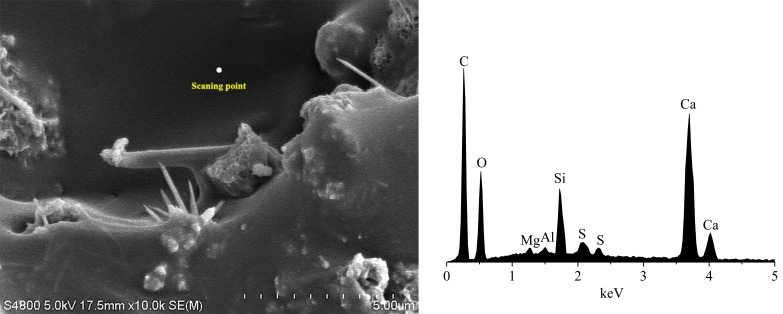
Chemical composition of CRME with 2% cement.

The asphalt emulsion mainly contains the elements of C, H, S, O, N. The cement mainly contains the elements of Ca, Si, Al, Fe, O. In order to identify the crystalline substance of CRME, an energy spectrum test of CRME mortar was conducted by the energy dispersive system of SEM PHILIPS-FEI Quanta 200. Figure 7 and Figure 8 and Table 4 show the results of energy spectrum analysis. The mortar without cement consists mainly of carbon. However, the element contents of O, Si, Ca increase and the element content of C decreases in the mortar with 2% cement. Meanwhile, the element of Al is found in energy spectrum in the CRME with 2% cement. The results indicate that the composite mortar is formed by hydration products and asphalt emulsion.

## 4. Conclusions

Performance and microstructure of CRME with different contents of cement were investigated in this paper. Based on the results and discussions, the following conclusions could be drawn:

(1) The high-temperature stability of CRME increases rapidly with the contents of cement from 0% to 3%. The moisture susceptibility of CRME increases rapidly with the contents of cement from 0% to 5%. The low-temperature cracking resistance of CRME gradually increased when the cement content is from 0% to 1.5%. However, it gradually decreased when the cement content is from 1.5% to 4%.
(2) The fracture surface and the chemical composition of CRME without cement and with different contents of cement are different by the SEM test and energy spectrum analysis. Hydration products are coated by asphalt emulsion when the content of cement is 1% and 2%. And the composite structure is formed by hydration products and asphalt emulsion.
(3) According to the results of the microstructure and performance tests, the optimum content of cement is suggested to be 1% to 2%.

## Figures and Tables

**Table 1:** Properties of asphalt emulsion.

Property	Test Result
Remaining amount on 1.18 mm sieve/wt.%	0.024
Residue content/wt.%	64.0
Penetration (25 °C, 100 g)/0.1 mm	68.1
Softening point/°C	45.1
Ductility (15 °C)/cm	76.5
Solubility in trichloroethylene/wt.%	99.1
Storage stability at 1 d/wt.%	0.6
Storage stability at 5 d/wt.%	2.7

**Table 2:** Gradations of RAP.

Size/mm	26.5	19	16	13.2	9.5	4.75	2.36	1.18	0.6	0.3	0.15	0.075
Passing Rate/%	100	100	97.6	94.9	86.7	56.7	35.1	22.6	14.0	6.7	4.8	4.2

**Table 3:** Gradations of new aggregates.

Size/mm		26.5	19	16	13.2	9.5	4.75	2.36	1.18
Passing rate/%	16–19	100	93.1	32.5	0.5				
	13.2–16	100	99.7	85.2	32.7	1.0	0.1		
	9.5–13.2	100	100	99.7	90.5	9.0	0.2		
	2.36–4.75	100	100	100	100	100.0	96.7	11.3	2.6

**Table 4:** Chemical composition of CRME with 0% and 2% cement.

Element		C	O	Si	Ca
0% cement	Weight percentage/%	89.56	5.74	1.01	2.35
	Atomic percentage/%	93.75	4.51	0.45	0.74
2% cement	Weight percentage/%	30.56	51.03	7.56	4.27
	Atomic percentage/%	40.48	50.57	4.27	3.84

## References

[1] Lin J., Hong J., Xiao Y. (2017). Dynamic characteristics of 100% cold recycled asphalt mixture using asphalt emulsion and cement. J. Clean. Prod..

[2] Sangiorgi C., Tataranni P., Simone A., Vignali V., Lantieri C., Dondi G. (2017). A laboratory and filed evaluation of Cold Recycled Mixture for base layer entirely made with Reclaimed Asphalt Pavement. Constr. Build. Mater..

[3] Ayar P. (2018). Effects of additives on the mechanical performance in recycled mixtures with bitumen emulsion: An overview. Constr. Build. Mater..

[4] Tataranni P., Sangiorgi C., Simone A., Vignali V., Lantieri C., Dondi G. (2018). A laboratory and field study on 100% Recycled Cement Bound Mixture for base layers. Int. J. Pavement Res. Technol..

[5] Brayton T.E., Lee K.W., Harington J., Kearney E.J. Construction and Materials Issues. Characterization of cold in-place recycling asphalt mixtures. Proceedings of the Construction Institute Sessions at the ASCE Civil Engineering Conference.

[6] Behnood A., Gharehveran M.M., Asl F.G., Ameri M. (2015). Effects of copper slag and recycled concrete aggregate on the properties of CIR mixes with bitumen emulsion, rice husk ash, Portland cement and fly ash. Constr. Build. Mater..

[7] Tebaldi G., Dave E.V., Marsac P., Muraya P., Hugener M., Pasetto M., Graziani A., Grilli A., Bocci M., Marradi A. (2014). Synthesis of standards and procedures for specimen preparation and in-field evaluation of cold-recycled asphalt mixtures. Road Mater. Pavement Des..

[8] Kandhal P.S., Mallick R.B. (1997). Pavement Recycling Guidelines for State and Local Governments Participant’s Reference Book (Report No. FHWA-SA-98-042).

[9] Niazi Y., Jalili M. (2009). Effect of Portland cement and lime additives on properties of cold in-place recycled mixtures with asphalt emulsion. Constr. Build. Mater..

[10] Hodgkinson A., Visser A.T. The role of fillers and cementitious binders when recycling with foamed bitumen or bitumen emulsion. Proceedings of the 8th Conference on Asphalt Pavements for Southern Africa (CAPSA’04).

[11] Kim Y., Lee H.D. (2012). Performance evaluation of Cold In-Place Recycling mixtures using emulsified asphalt based on dynamic modulus, flow number, flow time, and raveling loss. KSCE J. Civ. Eng..

[12] Godenzoni C., Graziani A., Bocci E., Bocci M. (2018). The evolution of the mechanical behaviour of cold recycled mixtures stabilised with cement and bitumen: Field and laboratory study. Road Mater. Pavement Des..

[13] Du S. (2015). Performance Characteristic of Cold Recycled Mixture with Asphalt Emulsion and Chemical Additives. Adv. Mater. Sci. Eng..

[14] Xu O., Wang Z., Wang R. (2017). Effects of aggregate gradations and binder contents on engineering properties of cement emulsified asphalt mixtures. Constr. Build. Mater..

[15] Lin J., Wei T., Hong J., Zhao Y., Liu J. (2015). Research on development mechanism of early-stage strength for cold recycled asphalt mixture using emulsion asphalt. Constr. Build. Mater..

[16] Ojum C.K. (2015). The Design and Optimisation of Cold Asphalt Emulsion Mixtures. Ph.D. Thesis.

[17] Du S. (2014). Effect of different fillers on performance properties of asphalt emulsion mixture. J. Test Eval..

[18] Modarres A., Nejad F.M., Kavussi A., Hassani A., Shabanzadeh E. (2011). A parametric study on the laboratory fatigue characteristics of recycled mixes. Constr. Build. Mater..

[19] Ma T., Wang H., Zhao Y., Huang X., Pi Y. (2015). Strength Mechanism and Influence Factors for Cold Recycled Asphalt Mixture. Adv. Mater. Sci. Eng..

[20] Xiao J., Yu Y. (2011). Research on moisture susceptibility of emulsion treated cold reclaimed asphalt mixture. Pav. Mater..

[21] Kavussi A., Nejad F.M., Modarres A. (2011). Laboratory fatigue models for recycled mixes with pozzolanic cement and bitumen emulsion. J. Civ. Eng. Manag..

[22] Modarres A., Ayar P. (2016). Comparing the mechanical properties of cold recycled mixture containing coal waste additive and ordinary Portland cement. Int. J. Pavement Eng..

[23] Dondi G., Mazzotta F., Sangiorgi C., Pettinari M., Simone A., Vignali V., Tataranni P. (2014). Influence of cement and limestone filler on the rheological properties of mastic in cold bituminous recycled mixtures. Sustainability, Eco-Efficiency, and Conservation in Transportation Infrastructure Asset Management.

[24] Recasens R.M., Pérez Jiménez F.E., Aguilar S.C. (2000). Mixed recycling with emulsion and cement of asphalt pavements. Design procedure and improvements achieved. Mater. Struct..

[25] Pérez I.P., Medina L., Del Val M. (2013). Ángel Mechanical properties and behaviour ofin situmaterials which are stabilised with bitumen emulsion. Road Mater. Pavement Des..

[26] Yan J., Leng Z., Li F., Zhu H., Bao S. (2017). Early-age strength and long-term performance of asphalt emulsion cold recycled mixes with various cement contents. Constr. Build. Mater..

[27] Kavussi A., Modarres A. (2010). Laboratory fatigue models for recycled mixes with bitumen emulsion and cement. Constr. Build. Mater..

[28] Wang Z.J., An D.D., Liu L., Wang H.F., Zhang Q. (2016). Quantitative evaluation of interfacial adhesion between cement emulsified asphalt mastic and RAP. J. Chang’an Univ. (Nat. Sci. Ed.).

[29] Ministry of Transport of the People’s Republic of China (2008). Technical Specifications for Highway Asphalt Pavement Recycling.

[30] Ministry of Transport of the People’s Republic of China (2007). Test Methods of Soils for Highway Engineering.

[31] Ministry of Transport of the People’s Republic of China (2011). Standard Test Methods of Bitumen and Bituminous Mixtures for Highway Engineering.

